# The Genetic Architecture of Juvenile Growth Traits in the Conifer *Torreya grandis* as Revealed by Joint Linkage and Linkage Disequilibrium Mapping

**DOI:** 10.3389/fpls.2022.858187

**Published:** 2022-06-27

**Authors:** Wenchong Chen, Weiwu Yu, Ang Dong, Yanru Zeng, Huwei Yuan, Bingsong Zheng, Rongling Wu

**Affiliations:** ^1^State Key Laboratory of Subtropical Silviculture, Zhejiang A&F University, Hangzhou, China; ^2^Center for Computational Biology, College of Biological Sciences and Technology, Beijing Forestry University, Beijing, China; ^3^Center for Statistical Genetics, Department of Public Health Sciences, Department of Statistics, The Pennsylvania State University, Hershey, PA, United States

**Keywords:** linkage map, QTL, linkage disequilibrium, linkage disequilibrium map, *Torreya grandis*

## Abstract

Despite its high economical and ornamental values, *Torreya grandis*, a dioecious non-timber coniferous species, has long been an underrepresented species. However, the advent and application of advanced genotyping technologies have stimulated its genetic research, making it possible to gain new insight into the genetic architecture of complex traits that may not be detected for model species. We apply an open-pollination (OP) mapping strategy to conduct a QTL mapping experiment of *T. grandis*, in which nearly 100 unrelated trees randomly chosen from the species’ natural distribution and their half-sib progeny are simultaneously genotyped. This strategy allows us to simultaneously estimate the recombination fractions and linkage disequilibrium (LD) coefficients between each pair of markers. We reconstruct a high-density linkage map of 4,203 SNPs covering a total distance of 8,393.95 cM and plot pairwise normalized LD values against genetic distances to build up a linkage-LD map. We identify 13 QTLs for stem basal diameter growth and 4 QTLs for stem height growth in juvenile seedlings. From the linkage-LD map, we infer the evolutionary history of *T. grandis* and each of its QTLs. The slow decay of QTL-related LDs indicates that these QTLs and their harboring genomic regions are evolutionarily relatively young, suggesting that they can better utilized by clonal propagation rather than through seed propagation. Genetic results from the OP sampling strategy could provide useful guidance for genetic studies of other dioecious species.

## Introduction

Most plant traits including those of agronomic importance are complex quantitative traits in nature, which are jointly controlled by an interacting network of genes, each with a small effect, and environmental factors (Lynch and Walsh, 1998). There is no exception with tree species. These genes underlying a complex trait are called quantitative trait loci (QTLs) or quantitative trait nucleotides (QTNs; Lynch and Walsh, 1998; Wu and Lin, 2006). Quantitative models have been established to separate the genetic and environmental effects for complex traits at the QTL level (Lander and Botstein, 1989; Yang et al., 2006; Wu et al., 2007; Li and Sillanpää, 2013, 2015; Yin et al., 2015; Zeng et al., 2019).

Linkage analysis based on a family-based design is a very popular approach for identifying and mapping QTLs (Wu et al., 2007; Sajjad et al., 2014). This approach makes use of allelic segregation and transmission at different loci to construct a genetic linkage map from a large number of molecular markers. A number of mapping examples using linkage analysis have been reported in backcross, double haploid, recombinant inbred lines, near isogenic line, and F_2_ populations, initiated with two inbred lines (Collard et al., 2005; Shar et al., 2020; Wang et al., 2020; Zhou et al., 2020). For forest trees, in which no inbred lines are available due to their long-life cycle, large genomes, and high heterozygosity (White et al., 2007), linkage analysis is conducted on the basis of a full-sib family derived from two heterozygous parents (Maliepaard et al., 1997; Wu et al., 2002b; Lu et al., 2004). The limitation of linkage analysis lies in its incapacity to high-resolution map QTLs, unless an extremely large number of samples sizes that is hardly met in forest genetics are used.

Apart from linkage mapping, there is an alternative mapping approach based on linkage disequilibria (LD). This LD-based approach can capitalize on recombinant events accumulated over evolutionary history and, therefore, provide high-resolution mapping of QTLs (Flint-Garcia et al., 2005; de Oliveira et al., 2010; Sajjad et al., 2014). Also, by sampling unrelated individuals from domesticated or natural populations, this approach can map a wide spectrum of allelic variants, including multiple alleles (Ersoz et al., 2007). There have been many examples of using LD analysis to map QTLs in forest trees; for example, QTLs detected for wood density in teak (*Tectona grandis*) (Vaishnav and Shamim, 2019) and the *LACCASE* gene (an important regulatory gene for lignin biosynthesis) characterized for Japanese larch (*Larix kaempferi*) (Liu et al., 2017). However, its application may be impaired by spurious LD detection and the occurrence of rare alleles increasingly recognized as important contributors of genetic variation (Cardon and Palmer, 2003).

To overcome the limitations of linkage and LD mapping, a joint linkage-LD analysis has been developed (Wu and Zeng, 2001; Wu et al., 2002a; Myles et al., 2009; Bennett, 2010; Lu et al., 2010; Pagny et al., 2012), which has been shown to increase the precision of QTL mapping and decrease its false positive rate. Unlike autogamous plants, forest trees mostly perform allogamous pollination, i.e., the pistils of a tree receive the sperms in the pollens randomly from different trees to fertilize their eggs. Taking advantages of this open pollination (OP) mating behavior, Wu and Zeng (2001) and Wu et al. (2002a) proposed an OP mapping strategy that combines the advantages of linkage mapping and LD mapping. The merit of the OP mapping strategy is augmented by its additional value to infer the genetic diversity of natural populations and the evolutionary history of QTLs (Sun et al., 2015; Zhu et al., 2015a,b). More recently, Zeng et al. (2019) have equipped this strategy with a capacity to estimate and test QTLs through non-DNA sequence-based (non-genetic) maternal inheritance.

Because of these unique advantages, also stimulated by the development of sequencing techniques that make it possible to genotype a massive amount of SNP markers even for un-sequenced forest trees (Wang et al., 2011; Huang et al., 2015), the OP strategy shows its increasing usefulness in QTL mapping. In this study, we use this strategy to map growth QTLs for *Torreya grandis* Fort. ex Lindl, an underrepresented dioecious gymnosperm species distributed in the southeastern China (Kang and Tang, 1994). As an economically important species producing edible nuts, *T. grandis* has been cultivated for more than 1000 years, but its systematic population and quantitative genetic study has not begun until very recently. Using the OP seeds from a single tree of *T. grandis* “Merrillii,” a commercial variety of *T. grandis*, Zeng et al. (2014) constructed a first low-density genetic map to which QTLs affecting juvenile growth traits were located. A second low-density linkage map of *T. grandis* was constructed using the OP design in which both parents and offspring were genotyped for the same set of markers (Zhu et al., 2015a). Using this linkage map, Zeng et al. (2019) further map QTLs that display transgenerational inheritance or epigenetic expression. Although these previous studies demonstrate the power of OP design to dissect complex traits in recalcitrant coniferous species, use of less expensive dominant markers, i.e., sequence-related amplified polymorphism (SRAP) and amplified fragment length polymorphism (AFLP), limits the coverage of the linkage maps reconstructed.

In this article, we report a large-scale OP-based mapping study by genotyping about 100 maternal *T. grandis* trees and 10 offspring of each maternal by genotyping-by-sequencing (GBS). After quality control, 70,580 SNP markers are detected to be segregating simultaneously in parental and offspring populations. By germinating OP seeds of each sampled maternal tree into seedlings, we implement the mapping algorithm of OP design (Wu and Zeng, 2001; Zhu et al., 2015a,b) to reconstruct a linkage map and identify QTLs on the map that govern stem growth of juvenile *T. grandis* trees. We further infer the evolutionary history of growth QTLs detected in *T. grandis*, providing new insight into the genome structure and organization of this less studied but important species.

## Materials and Methods

### Experimental Design and Sampling

Based on the Wu and Zeng’s (2001) OP mapping strategy, we collected seeds and leaves from 96 unrelated trees randomly distributed in a natural population of *T. grandis* in Xiaorong Village, Chenkan Town, Huizhou District, Huangshan City, Anhui Province of China (29°57′ N and 118°14′ E) in 2017. The leaves were used to isolate DNAs used to genotype sampled parental trees. The seeds were stratified in a greenhouse on campus of Zhejiang A&F University (30°15′ N and 119°43′ E). In the following spring (2018), germinated seeds were transplanted into a 22 cm × 21 cm (diameter × height) plastic pot to grow seedlings. Young leaves from seedling were collected for DNA isolation used to genotype the progeny. Seedling height and basal diameter growth was measured for each progeny from each parental tree at the end of each year for 2018–2021. These growth traits were used for QTL mapping.

Leaf samples from both 96 maternal trees and their 878 progeny (4–10 seedlings from each maternal tree) were sent to LC-Bio Technologies (Hangzhou) Co., Ltd., Hangzhou, China. These trees were genotyped for genome-wide SNPs by a GBS-based genotyping technique combining *Eco*RV and *Sca*I and SNP calling. An insert size of 430–460 bp was selected by gel electrophoresis for further sequencing library construction. Barcoded adaptors and common adaptors were then ligated to digested fragments, followed by every 96 random samples pooling and PCR amplification, in which only those short samples featuring a barcode + common adaptor combination were amplified. Finally, the fragments were enriched by PCR amplification and purified by magnetic beads. The use of a pair of SNPs for joint linkage and linkage disequilibrium analysis requires the information of the markers that are co-segregating in both parental and offspring populations. Excluding rare allele markers in the parental population, markers deviating from Mendelian segregation pattern in the offspring population, and missing markers (missing at >5%) in the parental population, there are 70,580 such quality SNP markers used for consequent analysis.

### Linkage Map Construction and Linkage Disequilibrium Estimation

A statistical algorithm for simultaneously estimating the recombination fractions and linkage disequilibria between each pair of markers using the OP design was described in detail by Wu and Zeng (2001), Wu et al. (2002a), and Zhu et al. (2015a,b). To help the readers understand this algorithm, we outlined its basic principle. Consider a pair of biallelic SNP markers that are co-segregating in parental population. Let S_*p*_ denote parental genotypes at these two markers. The pattern of marker co-segregation is determined by population genetic parameters (P); i.e., four haplotype frequencies, or equivalently, by allele frequencies at each marker and the LD between the two markers. The co-segregating marker pair is co-transmitted from parents to their half-sib progeny, forming offspring genotypes (denoted by S_*o*_). The pattern of co-transmission is determined by the recombination fraction, denoted by r. The likelihood of population genetic parameters and the recombination fraction given observed marker data is formulated as:


(1) $$L(P,r|S{}_{p},S{}_{o})=L{}_{p}(P|S{}_{p})+L{}_{q}(r|S{}_{p},S{}_{o},P)$$

which includes two components, the likelihood of population genetic parameters [L_*p*_(P| S_*p*_)] given parental marker genotypes and the likelihood of the recombination fraction [L_*q*_(r| S_*p*_, S_*o*_, P)] given parental and offspring genotypes and the estimates of population genetic parameters. Maximizing the likelihood in Eq. (1) is equivalent to maximizing L_*p*_(P| S_*p*_) and L_*q*_(r| S_*p*_, S_*o*_, P), individually. Wu and Zeng (2001) proposed the EM algorithm to jointly solve these two likelihoods. Specifically, L_*p*_(P| S_*p*_) allows us to estimate haplotype frequencies, from which allele frequencies and LD are estimated. The estimated LD was normalized as marker-marker correlation (r^2^), whereas L_*q*_(r| S_*p*_, S_*o*_, P) allows us to estimate the recombination fraction. Thus, for the same pair of markers, we can estimate both their recombination fraction and LD, which allows the linkage-LD map to be built.

We implemented Matlab R2019a to solve the likelihood of Eq. (1). The estimates of pairwise recombination fractions are used to reconstruct linkage maps. Optimal linkage groups were determined by changing the threshold of the recombination fraction and LOD. Markers of each group were ordered by using sum of adjacent recombination frequencies (SARF; Buetow and Chakravarti, 1987). The genetic distance between markers expressed as centiMorgan (cM) was calculated by transforming the recombination rate through the Kosambi mapping function. The R 4.0.3 package LinkageMapView (Ouellette et al., 2018) was adopted to draw the genetic linkage map and the marker density map.

### Quantitative Trait Loci Identification

Wu et al. (2002a) extended Wu and Zeng’s (2001) OP design to map QTLs for phenotypic traits. Consider a SNP with three genotypes AA, Aa, and aa which are segregating in the parental (maternal) population. Through OP, each maternal genotype combines with three possible paternal genotypes AA, Aa, and aa from the pollen pool, producing different proportions of offspring genotypes AA, Aa, and aa. The total number of each of these three offspring genotypes is the sum of the number of the offspring genotype produced by each maternal genotype, weighted by the frequencies of maternal genotypes. Let *n*_1_, *n*_2_, and *n*_3_ denote the total number of offspring genotypes AA, Aa, and aa, respectively, and let *y*_*i*_ denote the phenotypic value of a trait measured for an offspring individual. The likelihood of trait value at this SNP from the offspring population is formulated as:


(2) $$L(y)=\Pi {}_{i}{}_{=}{}_{1}{}^{n_{1}}f{}_{1}(y{}_{i})\Pi {}_{i}{}_{=}{}_{1}{}^{n_{2}}f{}_{2}(y{}_{i})\Pi {}_{i}{}_{=}{}_{1}{}^{n_{3}}f{}_{3}(y{}_{i})$$

where *f*_*j*_(*y*_*i*_) is the normal distribution density function with mean μ_*j*_ (*j* = 1 for AA, 2 for Aa, and 3 for aa) and variance σ^2^.

After the model parameters are estimated, a hypothesis test is performed to test whether there are significant QTLs affecting the growth trait. The null hypothesis that assumes no existence of a QTL, whereas the alternative hypothesis assumes its existence, is expressed as:


(3) H:0μ=1μ=2μ3

H_1_: at least one of the equations H_0_ above does not hold.

Under each hypothesis, we calculate its likelihood value, L_0_ for H_0_ and L_1_ for H_1_. The likelihood ratio (LR) is calculated by:


(4) LR=-2(L-0L)1

Permutation tests were conducted to calculate the critical threshold for testing significant QTLs (Doerge and Churchill, 1996). We estimated the proportion of phenotypic variance explained by each QTL.

## Results

### Genetic Linkage Map

**FIGURE 1:**
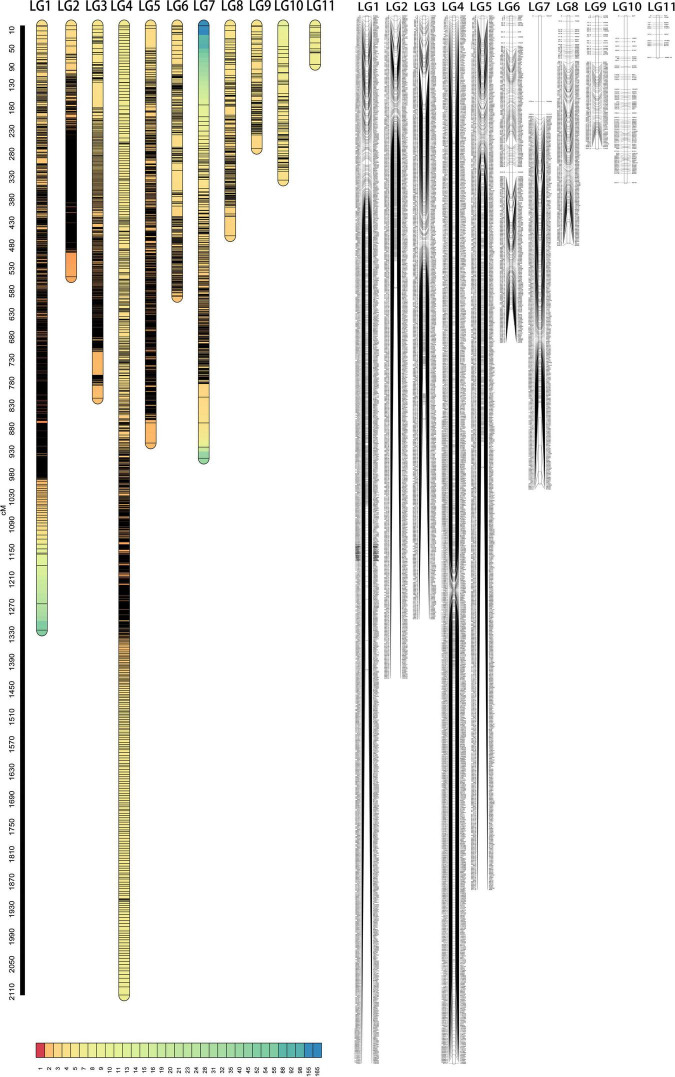
The marker density map **(left)** and linkage map **(right)** of *T. grandis* composed of 11 linkage groups (LG).

The offspring (seed) genotype of a maternal parent derives from the combination of its maternal gametes and the paternal gametes from the pollen pool. Thus, based on the segregation and recombination of two SNPs from a maternal genotype to its offspring, we can estimate the recombination fraction for such a SNP pair. By estimating pairwise recombination fractions, we reconstruct a high-density linkage map that contains 11 linkage groups (LG) based on the criterion of LOD (logarithm of the odd) >5. This map, composed of 4,203 SNPs and covering an overall map length of 8,393.95 cM with an average map distance of 2.00 cM, represents one of the highest marker density and genome coverage for *T. grandis* (Figure 1). The LG constructed ranges in length from 84.28 cM for LG11 with only 13 SNP markers to 2,117.02 cM for LG4 with 835 markers. LG1 has the largest number of markers, which is 903, LG2 has the smallest average distance of 1.09 cM, and LG7 has the largest gap of 172.93 cM (Table 1). The map has large gaps at several regions, such as those at the end of LG1 and both ends of LG7 (Figure 1).

**TABLE 1:** Statistical information of genetic linkage groups in *T. grandis*.

LG	Total number of markers	Total distance/cM	Average distance/cM	Max gap/cM
LG1	903	1,320.00	1.46	83.75
LG2	504	548.16	1.09	53.11
LG3	457	813.39	1.78	55.83
LG4	835	2,117.02	2.54	17.76
LG5	664	911.44	1.37	44.08
LG6	230	591.34	2.57	44.62
LG7	287	944.61	3.29	172.93
LG8	159	458.73	2.89	42.52
LG9	83	267.84	3.23	29.36
LG10	68	337.13	4.96	45.22
LG11	13	84.28	6.48	24.92
Total	4,203	8,393.95		

### Linkage-Linkage Disequilibrium Map

**FIGURE 2:**
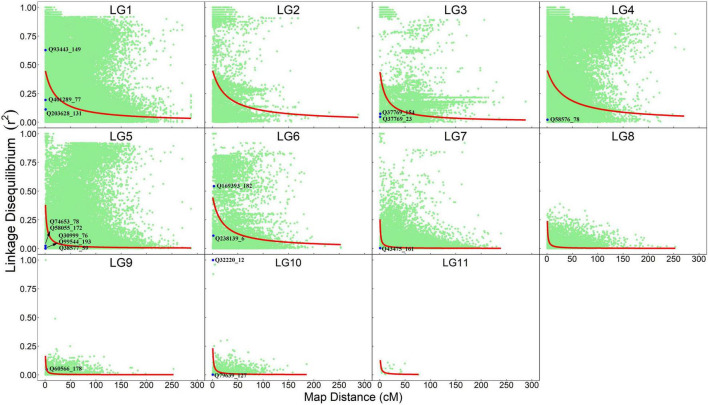
Linkage-linkage disequilibrium map expressed as a plot of normalized LD values against map distance for marker pairs from the same linkage groups. Red line denotes the decay curve of LD with increasing genetic distance. QTLs that are detected to affect seedling growth traits are indicated.

The information about how a pair of markers is co-segregating in natural populations is contained in maternal and paternal gametes, which can be extracted from the OP design. We estimate pairwise LD and plot their normalized values against map distances separately for each linkage group (Figure 2). As expected, LD decays with genetic distance, but with the degree of decay depending on linkage group. In general, linkage groups LG1–LG4 and LG6 have LD values that decay dramatically at a genetic distance of 10–20 cM; i.e., the decay of LD from 0.50 to 0.25 occurs at the genetic distance of 17.62 cM for LG1, 23.06 cM for LG2, 9.56 cM for LG3, 28.17 cM for LG4, and 15.89 cM for LG6. Yet, a dramatic LD decay in LG5 and LG7–LG11 occurs at a much shorter interval, i.e., 0.5–3.0 cM; i.e., the decay of LD from 0.50 to 0.25 occurs at a genetic distance of 2.11 cM for LG5, 0.52 cM for LG7, 0.51 cM for LG8, 0.54 cM for LG9, 0.66 cM for LG10, and 1.06 cM for LG11. Within the same linkage group, some marker pairs have large LD, but are separated by long genetic distances, suggesting that these markers may be subject to some recent evolutionary forces. Some markers have tight linkage and strong non-random association, implying that they have experienced a long evolutionary past.

Linkage disequilibrium between unlinked markers will disappear rapidly after several generations of random mating. Thus, the occurrence of such LD implies that relevant markers are experiencing evolutionary actions. We find that significant LD between markers from different linkage groups occurs, but they are not evenly distributed among linkage groups (Table 2). Markers on LG1 tend to be associated with those from LG2 to LG7. This finding, plus the facts that a good proportion of markers (8%) on LG1 has significant LD and that the frequencies of LD between all other linkage groups are very low, suggests that LG1 are more likely to be subject to recent evolutionary forces than other linkage groups.

**TABLE 2:** The number (upper diagonal) and proportion (lower diagonal) of marker pairs with significant LD within and between linkage groups (LG).

	LG1	LG2	LG3	LG4	LG5	LG6	LG7	LG8	LG9	LG10	LG11
LG1	31028 (0.08)	25251	21312	45807	39371	11046	16520	2857	2608	2448	273
LG2	0.08	4010 (0.03)	4334	7340	8189	4490	3412	574	931	849	7
LG3	0.07	0.01	1331 (0.01)	4455	9181	3807	4109	1043	1150	1087	82
LG4	0.15	0.02	0.01	6350 (0.02)	20549	9115	8693	1245	1745	1344	63
LG5	0.13	0.03	0.03	0.07	17232 (0.08)	8824	16101	3795	2642	2383	510
LG6	0.04	0.01	0.01	0.03	0.03	1072 (0.04)	3878	612	447	540	46
LG7	0.05	0.01	0.01	0.03	0.05	0.01	3784 (0.09)	1557	1143	1208	165
LG8	0.01	0.00	0.00	0.00	0.01	0.00	0.01	482 (0.04)	580	460	101
LG9	0.01	0.00	0.00	0.01	0.01	0.00	0.00	0.00	151 (0.04)	279	90
LG10	0.01	0.00	0.00	0.00	0.01	0.00	0.00	0.00	0.00	138 (0.06)	35
LG11	0.00	0.00	0.00	0.00	0.00	0.00	0.00	0.00	0.00	0.00	2 (0.03)

*On diagonal are the number and proportion (in parentheses) of significant marker pairs on individual linkage groups.*

### Quantitative Trait Loci Identification

**FIGURE 3:**
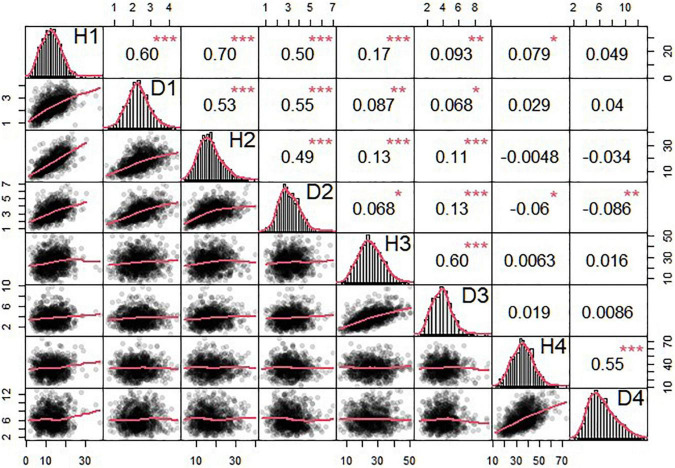
Phenotypic data analysis of seedling height and diameter in *T. grandis.* The triangle in the upper part of the figure is the correlation and significance and that in the lower part of the figure is scatterplot and its fitting curve. The diagonal part is normal distribution of seedling height and diameter data. 1, 2, 3, and 4 in the diagonal part means the year 2018, 2019, 2020, and 2021. *p < 0.05, **p < 0.01, ***p < 0.001.

The OP design can map QTLs to particular genomic regions of 11 linkage groups through a joint linkage-LD analysis. We consider two growth traits, stem height and basal diameter, measured for *T. grandis* seedlings from OP progeny in years 2018, 2019, 2020, and 2021. These two traits are found to vary considerably among progeny (Table 3 and Figure 3), implying the existence of their underlying QTLs. We identify 4 QTLs for stem height in 2018 and 2020 located on LG1, LG5, and LG7, and 13 QTLs for basal diameter in 2020 and 2021 on LG1, LG3, LG4, LG5, LG6, LG9, and LG10, respectively. Table 4 provides the information on the marker names of QTLs and their cross types and allele types. These QTLs mostly affect growth traits in an additive manner, but four of them display no dominant inheritance. Each QTL is found to account for a small portion of phenotypic variance, i.e., QTL heritability, suggesting that growth traits in *T. grandis* are polygenic.

**TABLE 3:** Variation analysis of phenotypic data in *T. grandis*.

Growth trait	Year	Max/mm	Min/mm	Mean/mm	Median/mm	SD*	CV/%
Seedling height	2018	408.0	11.0	125.6	125.0	4.76	37.90
	2019	436.0	36.0	167.9	163.0	5.71	34.01
	2020	504.0	58.0	238.5	231.0	7.32	30.69
	2021	735.0	112.0	350.5	348.0	9.40	26.82
Basal diameter	2018	4.67	0.54	2.24	2.22	0.63	28.13
	2019	7.35	1.08	3.02	2.95	0.85	28.15
	2020	9.79	1.21	3.86	3.82	1.13	29.27
	2021	12.96	1.82	6.26	5.90	2.11	33.71

*Standard deviation.

**TABLE 4:** Seedling growth-associated QTLs identified and their genetic effects on growth of different years in *T. grandis.*

Trait	Year	SNP position	QTL	Linkage group	Locus type	Allele	LR	Heritability	μ	*a*	*d*
Height	2018	869	Q30999_76*	LG5	Intercross	C/T	24.37	0.02	14.57	−1.94	−2.08
Height	2018	1519	Q43475_161	LG7	Intercross	G/A	23.94	0.02	13.21	−0.32	−1.86
Height	2018	3033	Q203628_131	LG1	Intercross	A/T	24.16	0.01	11.13	−0.75	1.47
Height	2020	2117	Q74653_78	LG5	Intercross	G/A	26.32	0.03	26.23	−1.34	−2.89
Diameter	2020	2312	Q93443_149	LG1	Testcross	C/T	20.67	0.02	3.86	0.41	NA
Diameter	2021	2854	Q169393_182	LG6	Testcross	G/A	19.85	0.02	5.92	0.60	NA
Diameter	2021	3224	Q238139_6	LG6	Testcross	T/C	19.90	0.02	6.36	−1.10	NA
Diameter	2020	1278	Q38577_59	LG5	Intercross	T/C	29.72	0.00	4.15	−0.15	−0.27
Diameter	2020	1925	Q60566_178	LG9	Intercross	G/A	38.92	0.03	4.12	−0.31	−0.10
Diameter	2020	2371	Q99544_193	LG5	Intercross	C/T	29.00	0.02	3.46	0.47	0.65
Diameter	2021	959	Q32220_12	LG10	Intercross	T/C	32.91	0.03	6.53	−0.50	−0.35
Diameter	2021	1217	Q37769_23	LG3	Intercross	G/C	37.22	0.04	6.49	−0.58	−0.26
Diameter	2021	1219	Q37769_154	LG3	Intercross	C/T	33.30	0.03	6.49	−0.54	−0.28
Diameter	2021	1882	Q58055_172	LG5	Intercross	A/G	35.48	0.05	6.85	−0.65	−0.77
Diameter	2021	1889	Q58576_78	LG4	Intercross	A/C	33.37	0.05	6.59	0.57	−0.46
Diameter	2021	2196	Q79639_127	LG10	Intercross	C/T	33.11	0.05	6.64	0.60	−0.45
Diameter	2021	4130	Q401289_77	LG1	Intercross	C/G	34.68	0.04	6.04	0.94	NA

*30999 is a marker tag and 76 is the location in this tag. LR, likelihood ratios; μ, phenotypic mean; a, additive effect; d, dominant effect; and NA, not detected.

By locating each of these QTLs on the linkage-LD map, we can infer their evolutionary history (Figure 2). The LD between two loci decays with generation at a rate proportional to their genetic distance. In general, if there is no linkage, disequilibrium will disappear at 5–6 generations of random mating. Yet, if there is strong linkage, LD will need a large number of generations to disappear. Thus, by comparing the size of both the linkage and LD, we can infer the number of generations that have passed since the production of disequilibrium. All QTLs, except for Q93443_149, Q169393_182 and Q32220_12, are located in the left bottom corner of the map, i.e., the markers associated with these QTLs have low linkage and disequilibrium values. Q93443_149, Q169393_182, and Q32220_12 reside in the region where there is little recombination but a remarkable disequilibrium.

## Discussion

The genetic mapping of complex traits is one of the most important topics in quantitative genetic research and plant breeding. Complex traits can be mapped by two approaches, linkage mapping and linkage disequilibrium (LD) mapping. Each of the two approaches has its own advantages and disadvantages in the accuracy, precision and power of QTL mapping and, thus, a simultaneous application of the two approaches has been considered in many genetic studies (Myles et al., 2009; Bennett, 2010; Lu et al., 2010; Pagny et al., 2012). Many of these studies simultaneously used linkage mapping and LD mapping for the same complex traits, but this simultaneous use was based on different mapping populations, i.e., controlled crosses for linkage mapping and founder-unknown populations for LD mapping. Although this can mutually validate mapping results from different approaches, such a simultaneous use does not resolve the accuracy and power issues characteristic of each approach. For example, the spurious detection of disequilibria may still occur for LD mapping.

To strengthen the advantages of each mapping approach and overcome disadvantages of them, they should be integrated into a unified framework for the same mapping population. Wu and Zeng (2001) and Wu et al. (2002a) are the first who have developed a joint model of linkage-LD mapping, validated the statistical properties of the model, and justified its practical application (Hou et al., 2009; Yin et al., 2015). In particular, based on the allogamous behavior of forest trees, they proposed an open-pollination (OP) sampling strategy for joint linkage-LD mapping, followed by elegant statistical algorithms for parameter estimation and testing. This OP mapping strategy not only preserves the merits of linkage mapping and LD mapping, but also generates a new value, i.e., it allows the cohesive integration of population genetics, evolutionary genetics, and quantitative genetics to understand the genetic diversity of populations and the evolution of QTLs. This strategy has found its biologically meaningful application for studying the genetics of Euphrates poplar (Zhu et al., 2015b) and *T. grandis* (Zhu et al., 2015a) and has been regarded as a generic tool for genetic mapping in forest trees and other outcrossing species (Sun et al., 2015). In this study, we employ Wu and Zeng’s (2001) OP design to map growth traits in *T. grandis*, an important but underrepresented tree species.

We simultaneously estimate the recombination fractions and LD coefficients between each pair of SNP markers genotyped for paternal trees and their half-sib offspring. By plotting normalized LD values against the recombination fractions, we chart a linkage-LD map, from which evolutionary events acting on the genome of *T. grandis* can be inferred. The slope of LD decay curve over genetic distances implies the evolutionary history of the species (Bennett and Binet, 1956; Sun et al., 2015). Previous studies suggest that LD values decays rapidly with genetic distance in coniferous trees (González-Martínez et al., 2007). Although this phenomenon is confirmed in this study, we gain additional insight into the genome structure of *T. grandis*. Our result shows that LD values in some narrow genomic regions decay dramatically, suggesting that these segments of the *T. grandis* genome may have experienced a long evolutionary history. We also find that some regions of the genome have been subjected to certain recent evolutionary forces, because large LD values are detected between genetically distant markers. A further investigation into the detailed distribution of genes located in evolutionarily old and young genomic regions is needed. Such information can help tree breeders choose an optimal breeding scheme for this coniferous tree species.

Ye et al. (2020) detected considerable differences in growth performance among *T. grandis* families, which is confirmed by this study. We implement Wu and Zeng’s (2001) algorithm to map QTLs for juvenile growth traits measured at different years in seedlings of half-sib offspring derived from paternal trees. We identified several stem height and basal diameter growth QTLs at different years, but did not find nay QTLs that are shared between different years. This finding, consistent with that detected from a linkage mapping experiment by Zeng et al. (2014), suggests that *T. grandis* activates diverse genetic systems in response to environmental change during its early establishment. This may also imply that this species preserves a rich warehouse of genetic variants to buffer against environmental perturbations in its growth and development.

Beyond traditional linkage mapping or LD mapping alone, the OP strategy allows us to infer the evolution of QTLs. We find that all QTLs, except for Q93443_149, Q169393_182, and Q32220_12, have weak associations with the markers that are highly linked with them, whereas Q93443_149, Q169393_182, and Q32220_12 are highly linked with the markers that are also strongly associated with them. Taken together, it is suggested that Q93443_149, Q169393_182, and Q32220_12 may still be relatively young in evolution, i.e., no adequate generations that have passed to lead LD to disappear, but the other QTLs may have experienced a long evolutionary history, because only an extremely large number of generations can make the LD of highly linked loci drop to a low level.

Our study can be methodologically improved at least in two aspects. First, growth is a dynamic process, but our mapping was based on growth traits measured at single time points. A dynamic functional mapping (FunMap) approach that integrates the mathematical principle of growth into a mapping context has been developed (Ma et al., 2002; Wu and Lin, 2006). Because of its biological relevance and statistical robustness, FunMap has been applied to many plant mapping projects (Yang et al., 2006; Wu et al., 2011; Li and Sillanpää, 2013; Camargo et al., 2018; Lyra et al., 2020), widely recognized as a powerful tool for QTL mapping (Li and Sillanpää, 2015). Second, our mapping study finds that each QTL explains a relatively small portion of genetic variance (<10%) in growth traits. Similar phenomena were also detected in other studies (González-Martínez et al., 2011). However, it is possible that a small-effect QTL may not be necessarily small, rather its independent effect is masked by the inhibition of negative regulators (Sun et al., 2021; Wang et al., 2021; Wu and Jiang, 2021). The exact effect of each QTL or gene can better be characterized through modeling gene-gene interactions (Wang et al., 2022). A new mapping approach, called functional graph theory (FunGraph) derived from FunMap, has been proposed to chart the genetic interactome network of complex traits from genetic association data (Dong et al., 2021; Feng et al., 2021). A detailed analysis of our *T. grandis* data from the OP mapping strategy deserves by FunMap and FunGraph.

## Data Availability Statement

The datasets presented in this study can be found in online repositories. The names of the repository/repositories and accession number(s) can be found in the article/supplementary material.

## Author Contributions

WC analyzed the data and drafted the manuscript. WY, YZ, HY, and BZ designed the experiment and collected the data. AD participated in data analysis. RW conceived the OP concept, revised the manuscript, and addressed the reviewer’s comments. All authors contributed to the article and approved the submitted version.

## Conflict of Interest

The authors declare that the research was conducted in the absence of any commercial or financial relationships that could be construed as a potential conflict of interest.

## Publisher’s Note

All claims expressed in this article are solely those of the authors and do not necessarily represent those of their affiliated organizations, or those of the publisher, the editors and the reviewers. Any product that may be evaluated in this article, or claim that may be made by its manufacturer, is not guaranteed or endorsed by the publisher.
